# Role of hepatic deiodinases in thyroid hormone homeostasis and liver metabolism, inflammation, and fibrosis

**DOI:** 10.1530/ETJ-22-0211

**Published:** 2023-04-13

**Authors:** Eveline Bruinstroop, Anne H van der Spek, Anita Boelen

**Affiliations:** 1 Department of Endocrinology and Metabolism, Amsterdam UMC, University of Amsterdam, Amsterdam, the Netherlands; 2 Amsterdam Gastroenterology Endocrinology Metabolism, Amsterdam, the Netherlands; 3 Department of Laboratory Medicine, Endocrine Laboratory, Amsterdam UMC, University of Amsterdam, Amsterdam, the Netherlands; 4 Amsterdam Reproduction & Development Research Institute, Amsterdam, the Netherlands

**Keywords:** thyroid, liver, deiodinases, metabolism, hepatitis, NAFLD

## Abstract

Thyroid hormones play an essential role in regulating whole-body homeostasis. Deiodinases are known to convert thyroid hormone from the prohormone thyroxine (T4) to the bioactive hormone tri-iodothyronine (T3) and convert both T4 and T3 toward their inactive metabolites 3,3’,5’-tri-iodothyronine (rT3) and 3,3’-di-iodothyronine (3,3’-T2). Deiodinases are thus important for the regulation of intracellular thyroid hormone concentrations. This is known to be crucial both during development and adult life in regulating thyroid hormone-related gene transcription. This review discusses the importance of liver deiodinases in determining serum and liver thyroid hormone concentrations, liver metabolism and liver disease.

## Introduction

### Deiodinases

The thyroid produces the thyroid hormone, mainly thyroxine (T4) and to a lesser extent tri-iodothyronine (T3). Within the liver, thyroid hormones are transported across the cell membrane by thyroid hormone transporters. The known transporters are members of the monocarboxylate transporter family (MCT8 and MCT10) and the organic anion-transporting polypeptide family (OATP1B1) (1). The prohormone T4 is most abundantly secreted by the thyroid. Therefore, peripheral conversion toward T3 is important as this is the major hormone activating the thyroid hormone receptor. For liver, the thyroid hormone receptor-β1 is most abundant, whereas in, for example, heart and bone, the thyroid hormone receptor-α1 is predominant.

The intracellular concentration of thyroid hormone is regulated by specific enzymes, so-called deiodinases (2). Deiodinases are able to remove an iodine atom from the outer or inner ring of the tyrosyl backbone. There are three types of deiodinases, type 1, type 2, and type 3. They are differentially expressed in tissues and have different functions in thyroid hormone deiodination. Deiodinases are important for determining intracellular thyroid hormone availability. Type 1 deiodinase (Dio1) is expressed in the liver, kidney, thyroid, and pituitary. Dio1 catalyzes outer and inner ring deiodination of T4, T3, and rT3, and, thus, can both activate and inactivate thyroid hormone. It was shown that rT3 and T3-sulfate are the preferred Dio1 substrates (3). Type 2 deiodinase (Dio2) is present in the brain, brown adipose tissue, heart, and pituitary. Dio2 produces T3 and due to a lower Km value for T4 compared to Dio1, it is catalytically much more active. By outer ring deiodination, it can also convert rT3 toward 3,3’-di-iodothyronine (3,3’-T2). Type 3 deiodinase (Dio3) terminates thyroid hormone action by inner ring deiodination of both T4 and T3 yielding rT3 and 3,3’-T2, respectively. Dio3 is highly expressed during development supposedly to protect tissues from high intracellular thyroid hormone levels. During adulthood, it is mainly expressed in the brain and placenta, and under physiological conditions, in low concentrations in other tissues such as the liver (4). Besides deiodination, sulfation by sulfotransferases and glucuronidation by UDP-glucuronosyltransferases are involved in the metabolism of thyroid hormone and its excretion via the bile and urine (5). This review will discuss the importance of liver deiodinases in health and disease.

### Liver deiodinases

In the liver, Dio1 is the most abundant deiodinase and is highly regulated by T3 itself via two thyroid hormone response elements in the human gene encoding Dio1 (6). In rats, reduced liver Dio1 activity is observed after thyroidectomy with approximately 30% of Dio1 compared to euthyroid rats. Conversely, increased liver Dio1 is found after T4 injection in rats (7, 8). Therefore, Dio1 expression and activity are currently the best indicators of hepatic T3 status. Interestingly, it was also shown that 3,5-di-iodothyronine (3,5-T2) is able to induce hepatic Dio1 activity in mice (9). In the healthy adult liver, there is no detectable Dio2 mRNA or activity. Liver Dio3, the thyroid hormone-inactivating enzyme, is expressed at low levels in a healthy adult liver and, however, may play a role during liver regeneration and liver disease (10).

**Figure 1:**
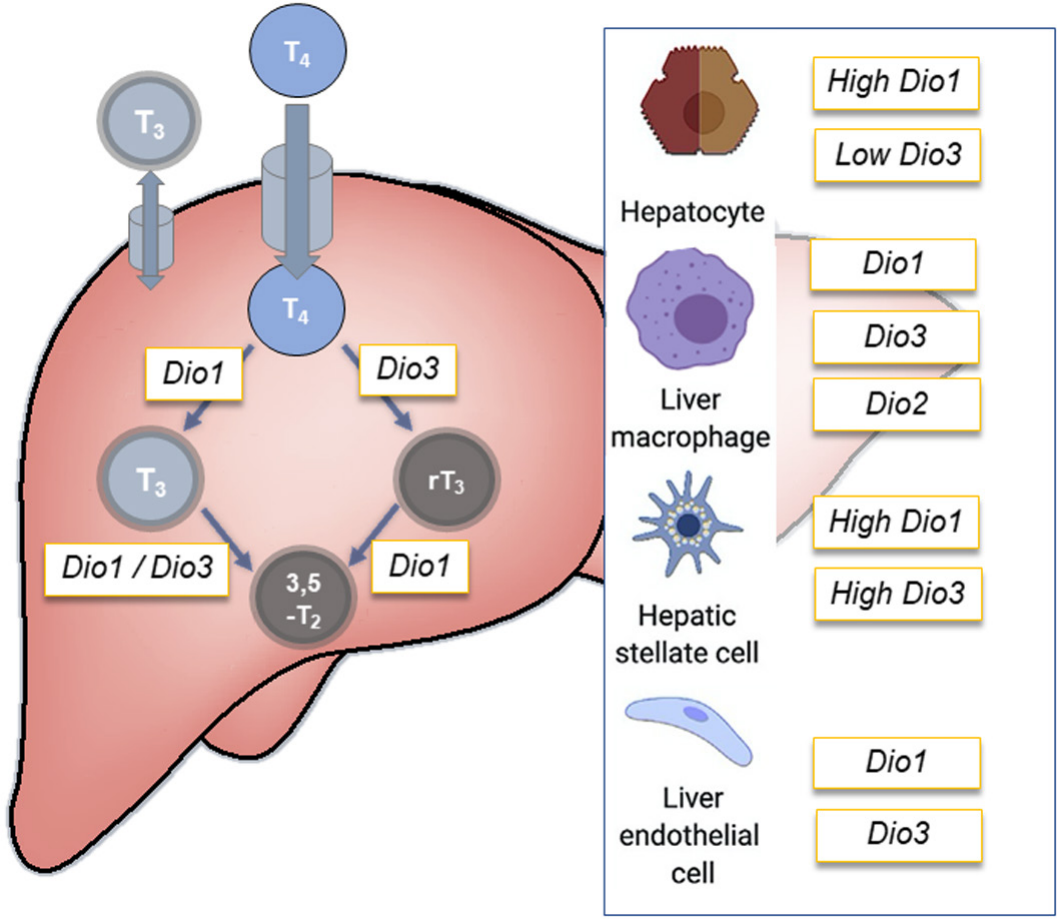
Overview of major thyroid hormone-metabolizing enzymes in the liver and their expression in different cell types.

**Figure 2:**
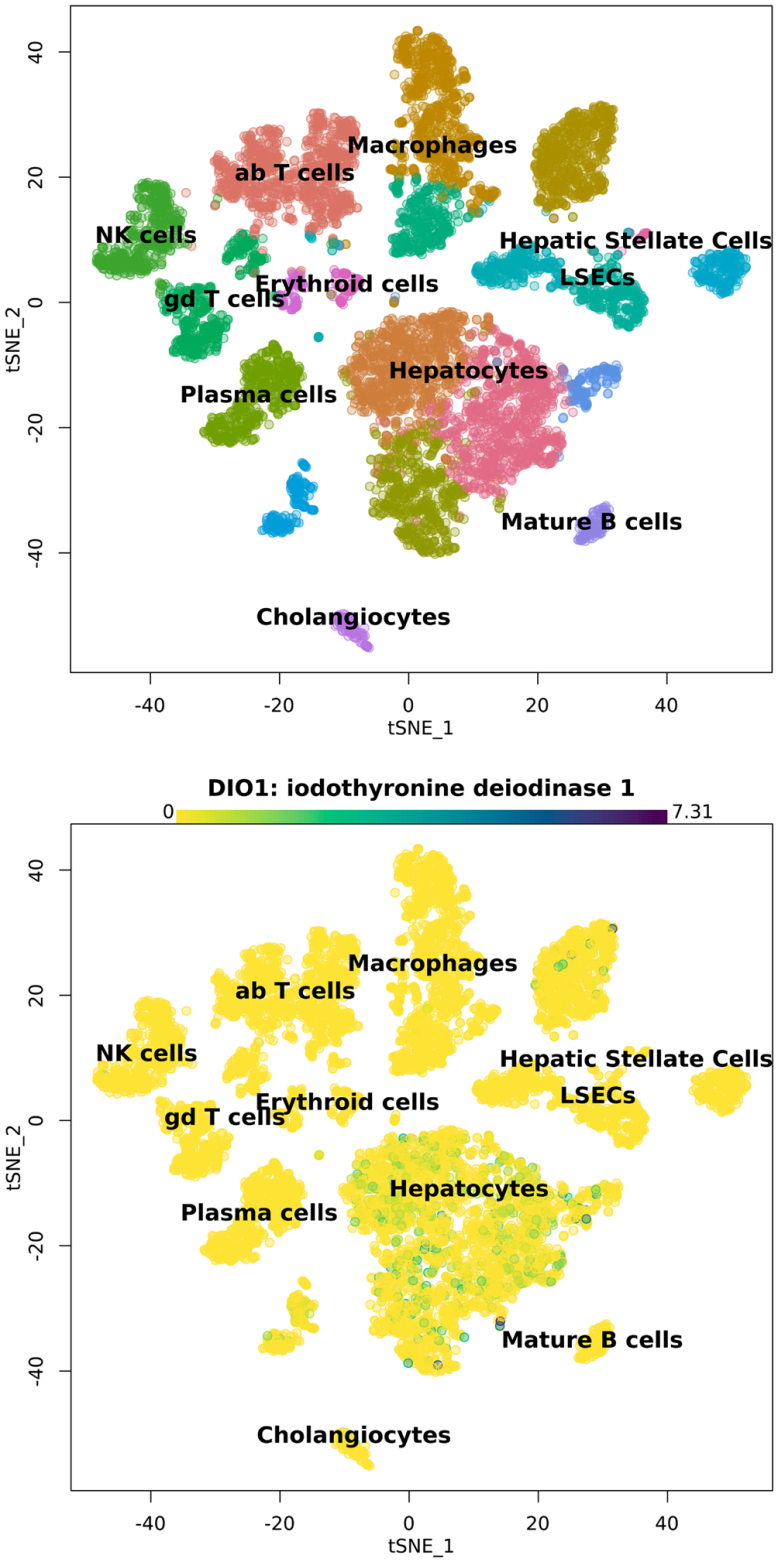
Expression profiles in human liver of Dio1 in different cell types of the liver from single-cell RNA sequencing of an open-access database (used app created by MacParland and colleagues (12) to make the cluster plots) (12).

When discussing the liver as a whole, however, we do not appreciate individual cell types within the liver differentially expressing the three deiodinases (Fig. 1). Bohinc and colleagues investigated the expression profiles of Dio1 and Dio3 in primary mouse hepatocytes, liver resident macrophages (Kupffer cells), liver fibroblasts (hepatic stellate cells), and liver sinusoidal epithelial cells (11). Dio1 mRNA expression was highest in hepatocytes followed by hepatic stellate cells. This was also observed in a human single-cell RNA sequencing experiment showing the expression of DIO1 mainly in hepatocytes and stellate cells (Fig. 2) (12). DIO1 mRNA expression was found in Kupffer cells and liver sinusoidal epithelial cells, albeit at significantly lower expression levels. Dio3 expression is much lower than Dio1 in the whole liver and most abundantly found in primary hepatic stellate cells (11). Lower expression was found in liver sinusoidal epithelial cells and Kupffer cells. Almost no Dio3 expression was found in hepatocytes. The low expression of Dio3 also translates to low Dio3 activity in the liver compared to hepatic Dio1 activity. Normal-fed rats display about 30 pmol/min/mg Dio1 activity in liver tissue whereas Dio3 activity is around 0.1 fmol/mg/min (13). In summary, Dio1 is the most abundant deiodinase in the liver mostly expressed in hepatocytes, however, also found in other cell types. Dio3 is hardly expressed, however, mostly in hepatic stellate cells and only little in hepatocytes.

Apart from different cell types, liver zonation also affects local T3 action differently along the porto-central axis of the liver units. It was shown that both TRβ and DIO1 protein expression increase further from the portal system with the highest concentration around the central vein (14).

## Role of liver deiodinases for systemic and liver T3 production

Traditionally, it is believed that most human T3 (80%) is produced by extra-thyroidal outer-ring deiodination of T4 in peripheral tissues with a major role for Dio1 in the liver. For rodents, this is shown to be lower estimated at 60% (15). However, the role of hepatic deiodinases in systemic T3 production is still under debate due to the studies performed on Dio1-deficient mice and humans carrying Dio1 mutations/polymorphisms (Table 1). Recently, the effect of liver-specific Dio1 knockdown and Dio1/Dio3 overexpression in the liver has been assessed (Table 2).

**Table 1:** Mouse and human studies investigating Dio1 inactivating and activating models.

Study	Model	Serum T4	Serum T3	Serum rT3
Schoenmakers *et al.* (1993) (17)	C3H mouse (inactivating)	⇑	=	⇑
Schneider *et al.* (2006) (19)	Mouse Dio1 knockout (inactivating)	⇑	=	⇑
Franca *et al.* (2021) (20)	Human Dio1 mutation (inactivating)	U	U	⇑
De Jong *et al.* (2007) (21)	Human Dio1 polymorphism (inactivating)	⇑	⇓	⇑
Panicker *et al.* (2008) (22)	Human Dio1 polymorphism (activating)	⇓	⇑ (*P* = 0.058)	⇓
De Jong *et al.* (2007) (21)	Human Dio1 polymorphism (activating)	=	⇑	=

T3, tri-iodothyronine; T4, thyroxine; U, unknown.

**Table 2:** Mouse models investigating liver-specific modulation Dio1 inactivating and activating models.

Study	Model	Serum T4/liver T4	Serum T3/liver T3	Serum rT3
Bruinstroop *et al.* (2021) (31)	Mouse Liver Dio1 knockdown (WDF)	⇑/⇑	=/=	U
Streckfuss *et al.* (2005) (30)	Mouse Liver selenoenzyme knockout	=/U	=/U	U
Yamauchi *et al.* (2022) (32)	Mouse Liver Dio1 overexpression	=/U	=/U	=

T3, tri-iodothyronine; T4, thyroxine; U, unknown.

### Serum thyroid hormone in Dio1 activating or inactivating models

Whole-body Dio1 modulation in mice also affects Dio1 expression in the kidney, pituitary, and thyroid. However, the liver is the largest Dio1 organ with also high Dio1 activity (16). The C3H/HeJ (C3H) inbred mouse was the first model to study the effects of low Dio1 activity as hepatic Dio1 activity is only 10% that of the C57J3L/6J (C57) inbred strain (17, 18). Later in time, a second model was introduced by generating whole-body Dio1 knockout mice (D1KO) mice with no Dio1 activity in the liver, kidney, and thyroid (19). In both models, a similar pattern of serum thyroid hormone levels was observed. Serum T3 and thyroid-stimulating hormone (TSH) concentrations remained within the normal range whereas serum T4 and rT3 concentrations were elevated.

In humans, two human DIO1 missense mutations (D1-N94K/D1-M201I) leading to reduced Dio1 activity have been described (20). These patients were found due to their increased rT3 concentrations, and no changes in serum T4 were observed. However, a DIO1 polymorphism (rs11206244) in the elderly population leading to reduced Dio1 activity showed that the increase of serum rT3 was also accompanied by lower T3 and higher fT4 concentrations (21). Conversely, two human polymorphisms have been described as leading to increased Dio1 activity (21, 22). Both polymorphisms did show increased serum T3 concentrations with only one polymorphism showing indeed decreased fT4 and rT3 concentrations (22). In conclusion, all whole-body Dio1 alterations in mice and humans but one human activating polymorphism have a clear effect on rT3 (Table 1). This shows that rT3 is the main substrate of Dio1 and indicates Dio1 as an rT3-clearing enzyme. The effects on T3 and the role of Dio1 as a T3-producing enzyme seem more variable. Interestingly, mainly the human polymorphisms show an effect on serum T3. This is probably due to the larger contribution of peripheral conversion in humans vs rodents (23). Furthermore, species differences in humans and mice exist between the half-life of T3 (humans T3 1 day, mouse T3 0.45 day; humans T4 5–9 days, mouse T4 0.5–0.75 days), and in mice, the high-affinity T4-binding globulin is absent. All these factors could contribute to the absent T3 phenotype in mice.

Interestingly, some studies point toward the hypothesis that the minor net effect of Dio1 on T3 is a result of the role of Dio1 both in T3 production and T3 clearance. This role of Dio1 in T3 status becomes more evident in the hyperthyroid state as it was shown that Dio1 is more involved in T3 production under these conditions. *In vitro*, the contribution of Dio1 in T3 production was higher than that of Dio2 during hyperthyroidism shown in an artificial model using transfection of human embryonic kidney (HEK-293) cells with human DIO1 or DIO2 (24). Whereas Dio2 activity is inversely related to fT4 concentration due to substrate-induced ubiquitination of the enzyme, Dio1 activity increased during hyperthyroidism (25). This is also supported by patients with Graves’ disease in which PTU, a selective inhibitor of Dio1 activity in all tissues (e.g. thyroid, liver, kidney), reduced T4 to T3 conversion by 50% (26). In athyreotic subjects with LT4 replacement aimed toward euthyroidism, only a 25% decrease in T4 to T3 conversion was observed when treated with PTU (27, 28). This increased production of T3 by Dio1 in hyperthyroidism has always been intriguing as it does not fit the classic feedback mechanism. Possibly this can be explained by the simultaneous role of Dio1 in T3 clearance. In both C3H mice and Dio1 KO mice, T3 injection results in a larger increase in serum T3 concentrations in these models compared to controls (19, 29). This indicates that Dio1 is also important in the clearance of T3. In Dio1 KO mice, radiolabeled T3 injection resulted in a marked decrease in urine excretion of T3. More than 80% of the T3 was now excreted in the feces as opposed to equal amounts in urine and feces in wild-type mice. This may indicate that although Dio1 contributes to a greater extent to the T3 production in hyperthyroidism, Dio1 also increases T3 clearance and is thus beneficial in maintaining serum T3 concentrations within normal limits in the hyperthyroid state.

Whether the effects of whole-body Dio1 mutations are primarily caused by liver Dio1 is intriguing. In a liver-specific model of reduced deiodinase expression by genetically inactivating hepatic selenoenzyme expression under the control of a hepatocyte promotor (albumin), a 90% decrease in Dio1 activity was found (30). In this model, TSH, total T4, and total T3 serum concentrations were not different compared to controls. This is opposed to the increased serum T4 levels observed in C3H and Dio1 KO mice. However, another model of liver-specific Dio1 shRNA knockdown (AAV8-albumin-mDio1-shRNAmir) did show increased serum T4 compared to control shRNA both on a western diet with fructose (31). Conversely, Yamauchi and colleagues overexpressed Dio1 specifically in the liver (pLIVE vector) (32). They first confirmed the higher activity of Dio1 after viral vector delivery by measuring higher T3 concentrations in the media of HEK293T cells after the addition of T4 as a substrate. When the Dio1 overexpression vector was delivered in mice, no changes in free T3 (fT3), free T4 (fT4), rT3, and TSH were measured after 2 and 7 days. These studies showed that in a liver-specific model of Dio1 modulation, no changes in serum T3 were observed and only in one study, serum T4 increased. Whether the major effects on serum rT3 and T4 are only caused by liver Dio1 remains unknown.

### Liver thyroid hormone concentration and action in Dio1/3 activating or inactivating models

Finally, the effects of Dio modulation on local liver thyroid hormone production and action are of interest. In Dio1 inactivating models, the question arises what happens to local T3 concentrations due to the proposed role of Dio1 both in T3 synthesis and clearance. Whole-body Dio1 KO mice even showed higher liver T3 concentrations (33). In liver-specific knockdown of Dio1 (Liver Dio1 KD) mice on a western diet with fructose showed no changes in liver T3 and increased liver fT4 is observed. Therefore, it may be hypothesized that also in the liver, both T3 production and clearance result in no or only minor changes in liver T3 concentrations. This is supported by a 77% reduction of deiodination activity with T3 as a substrate in Dio1 KO mice indicating an important role of liver Dio1 over Dio3 in T3 to 3,3’-T2 conversion (19). It may be interesting in future studies to distinguish between cytoplasmic and nuclear T3. It has been hypothesized that T3 produced by Dio1 may remain predominantly within the cytoplasm and equilibrate with the serum in contrast to Dio2, located in the endoplasmatic reticulum, which largely contributes to the local (cellular) T3 production (34). Therefore, it is interesting to investigate the effects of Dio1 inactivation on T3-related gene transcription. No changes in hepatic glycerol-3-phosphate dehydrogenase (*Gdp1*), glucose-6-phosphatase (*Gcpc*), and *Thrsp* were found between Dio1 KO mice and wild-type mice suggesting similar nuclear T3 availability in the liver (19). Interestingly, in the mice with reduced selenoenzyme expression affecting all liver deiodinases, T3 target genes *Thrsp* and *Gdp1* were reduced in Dio-deficient livers to about 60–70% of controls indicating that nuclear T3 availability may be impaired. However, mRNA and activity of the T3 target gene malic enzyme 1 (*Me1*) was significantly increased. Whether these changes in T3 target genes relate to Dio1 expression or the overall reduced selenoenzyme expression remains unanswered. In future experiments, it will be interesting to further investigate the role of liver Dio1 on T3 in stressed conditions such as hyperthyroidism and fasting/feeding conditions to investigate whether Dio1 contributes to local liver T3 production in these states. It does seem apparent that also T3 clearance is simultaneously affected.

Under physiological conditions, hepatic Dio3 expression and activity are very low. Yamauchi and colleagues overexpressed Dio3 specifically in the liver by viral vector delivery (pLIVE) (32). These constructs were only tested for their increased deiodination *in vitro* by transfecting HEK293 cells. Of note, deiodinase activity measurements were not performed with radioactive iodothyronines of the preferred substrates for which consensus has been reached and *in vivo* activity was not assessed (35). Taking this into consideration, they found *in vitro* no effect of Dio1 and Dio2 overexpression on serum thyroid hormones. However, Dio3 overexpression showed a clear phenotype on serum thyroid hormones by decreasing fT3 and more dramatically fT4 concentrations resulting in an increased fT3/fT4 ratio. Furthermore, serum rT3 concentrations were significantly increased. Gene expression of *Dio1, ldh3a,* and*Cyp17a1* was decreased whereas* Tapbp* expression was increased. This probably represents a decrease in fT3 and fT4. Whether this overexpression of Dio3 represents normal physiology is questionable; however, their phenotype on serum thyroid hormone concentrations is clear. It has been suggested that increased Dio3 during fasting also contributes to the decrease in serum T3 and T4 concentrations observed under these conditions. However, Galton and colleagues showed that serum thyroid hormone concentrations during fasting were not different in Dio3 KO mice compared to WT mice (36).

## Role of liver deiodinases in liver metabolism

**Figure 3:**
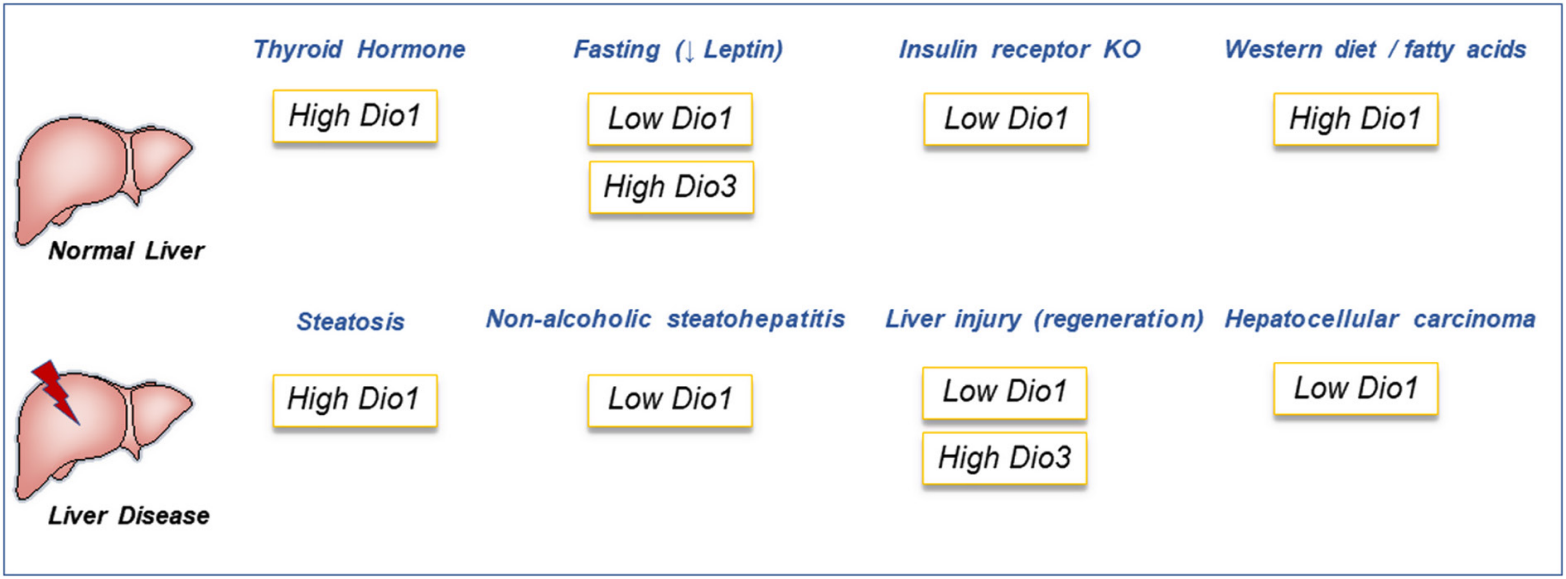
Overview of changes in Dio1 and Dio3 mRNA/activity during normal liver physiology and in liver disease.

Liver deiodinases are known to be affected in different metabolic states (Fig. 3). It is well established that (long-term) fasting lowers both serum T4 and T3 concentrations in humans and rodents. When mice were fasted for 36 h, liver T4 did not change compared to controls while liver T3 concentrations decreased (13). These changes were accompanied by decreased liver Dio1 mRNA and increased Dio3 mRNA and activity (13). The increased Dio3 activity was mediated via CAR activation and mTOR inhibition (37). Other studies have found a decrease in Dio1 activity after 30 days of 40% food restriction (38). Decreased Dio1 activity and increased Dio3 activity would result in reduced local T3 production and increased T3 clearance resulting in a reduction of intracellular T3 availability. However, it is unknown whether this intrahepatic regulation of deiodinases affects local T3 concentration and transcription or whether serum T3 levels remain the major determinant of hepatic T3 action. Lower liver T3 concentrations were accompanied by a reduced transcription of *Thrsp*, a T3-responsive gene (13). Interestingly, fasting in the Dio1 KO mouse resulted in increased rT3 concentrations due to reduced clearance of rT3 by Dio1 (36). Whether the changes in liver deiodinases are regulated by nutrients, hormones, or serum thyroid hormone levels is intriguing. Boelen and colleagues showed that the increase in Dio3 activity is regulated by the drop in leptin levels after fasting (39). In the fasted state, there are also low levels of circulating insulin. Liu and colleagues performed a knockdown of the insulin receptor in the liver by injecting 10-week-old InsR (fl/fl) (Lox) mice with an adenovirus carrying the Cre recombinase with the liver-specific albumin promoter (Ad-Cre) (33). This led to a 90% reduction of the liver insulin receptor. These mice also showed a marked reduction in Dio1 mRNA expression. This suggests that Dio1 might be downstream of insulin signaling or insulin-mediated nutrient flux.

It was also shown that Dio1 activity is responsive to nutrient excess (31). Gavin and colleagues showed that both a protein and carbohydrate diet increase T4 to T3 conversion in rat liver, whereas increasing glucose concentrations were more potent in this effect (40). Furthermore, mice fed a western diet with fructose (WDF diet) for 8 weeks showed increased hepatic Dio1 activity. The increase of Dio1 was due to a direct effect on hepatocytes as a combination of saturated and unsaturated fatty acids (oleic acid and palmitic acid) in the liver cell line, HepG2, increased Dio1 mRNA after 6 h of incubation (31). Xia and colleagues observed decreased Dio1 activity, *Dio1* mRNA, and DIO1 protein in obesity-prone mice after 7 weeks of high-fat diet opposed to increased Dio1 expression in obesity-resistant mice. It was predicted that *Dio1* was one of the targets of miR-383 in which an inverse relationship between *Dio1* mRNA and miR-383 was seen (41). In primary mouse hepatocytes, fatty acids increased miR-383 expression with a related decrease in *Dio1* mRNA expression. Induction of miR-383 by anti-miR-383 induced Dio1 expression and reduced intracellular triglycerides (41). The link between nutrients and Dio1 is still largely unknown; however, one might speculate a role for insulin-related nutrient-responsive liver transcription factors such as FOXA1 and FOXA2 (42).

There is evidence that these changes in Dio1 mRNA levels and/or activity are important in the regulation of liver metabolism. Liu and colleagues showed both in HepG2 cells (a human hepatocyte cell line) and *in vivo* that knockdown of Dio1 leads to reduced synthesis of apolipoprotein A-I (APOA-I) (33). ApoA-I, the major protein component of the anti-atherogenic lipoprotein HDL, is mainly present in the liver. The authors showed that intrahepatic T3 concentrations were not affected by this reduction of *Dio1* mRNA concentrations. Furthermore, insulin and Dio1 regulate the activity of a different promotor region of the *APOA1* gene than T3 does. The authors postulate that the effects of DIO1 on APOA-I are independent of T3. Bruinstroop and colleagues showed that prevention of the diet induced increase in Dio1 activity by Liver Dio1 KD increased liver triglycerides and liver total cholesterol (31). In addition, Dio1 KD in AML12 cells, a murine hepatocyte cell line, resulted in lower oxygen consumption rates (31). It was postulated that the early increase of Dio1 activity after a WDF diet may be compensatory to reduce the storage of fat within the liver. Although increased T4 concentrations were shown in the liver after a WDF diet, no changes were observed in liver T3 concentrations. Therefore, it is unknown whether the effects of Dio1 on fat accumulation in the liver are T3-related.

## Role of liver deiodinases in liver disease

The function of the normal liver can be altered by diseases involving liver inflammation (hepatitis), scarring (fibrosis), and cancer (hepatocellular carcinoma). Different cell types within the liver contribute to these disease states, mainly hepatocytes, Kupffer cells, hepatic stellate cells, and liver sinusoidal epithelial cells. It was shown in HepG2 cells that treatment with the pro-inflammatory cytokine IL1-beta reduces Dio1 enzyme and promotor activity (43, 44). In an animal model of liver fibrosis induced by a methionine- and choline-deficient (MCD) diet in rats, it was previously described that Dio1 activity is decreased (45). This MCD diet causes rapidly progressive steatohepatitis and liver fibrosis leading to reduced food intake and body weight loss. To investigate the change in Dio1 activity during the progression of non-alcoholic fatty liver disease (NAFLD) in a more obesogenic model, mice were fed a WDF diet. It was observed that in the early phase (8 weeks of diet) Dio1 activity is increased followed by a return to baseline after 24 weeks of WDF. The Dio1 activity was significantly and positively associated with the T3/T4 ratio. Although in non-alcoholic steatohepatitis (WDF 16 and 24 weeks) there is increased expression of pro-inflammatory cytokines (IL6, IL1-β, TNFα), *Dio1* mRNA does not decrease below the level of mice fed a normal chow diet after 24 weeks of diet (31, 46) This shows that in this obesogenic NAFLD model, the presence of inflammatory cytokines does not directly imply decreased deiodinase activity measured in whole liver; however, the diet-induced increase is dampened. It was recently shown that thyroid hormone given in mice with steatohepatitis induced by WDF is still able to induce *Dio1* mRNA levels about two-fold (46). The decrease in Dio1 during liver inflammation was also shown by the study by Bohinc and colleagues where they induced liver injury with a bile duct ligation in rats (11). On days 3, 7, and 14 after bile duct ligation, a dramatic decrease in Dio1 mRNA was observed together with a decrease in Dio1 protein measured by western blot and immunohistochemistry. Conversely, an increase in *Dio3* mRNA and protein levels was observed. Intrahepatic thyroid hormone concentrations (T3 and T4) were decreased, and positively regulated T3 target genes (*Thrsp* and *Me1*) were downregulated in this model. Immunohistochemistry revealed that during liver injury the major Dio1 expressing cell type was now a ductular type cell, as opposed to hepatocytes during normal conditions. Furthermore, Dio3 mainly accumulated in stromal cells and colocalized with markers for hepatic stellate cells (7). The injury-activated hepatic stellate cells play a pivotal role in liver regeneration. Liver injury induced sonic hedgehog (Shh) signaling increasing Dio3 in hepatic stellate cells. The Shh inducible transcription factor, Glioblastoma 2 (Gli2), has been shown to directly interact with the *Dio3* promoter to induce *Dio3* expression (47). Disruption of Shh signaling during liver injury by conditionally deleting Smoothened (*Smo*), an obligate component of the canonical Shh pathway, in myofibroblasts after bile duct ligation prevented the increase in *Dio3* mRNA. Furthermore, the reduction in *Dio1* expression was partly prevented indicating that this decrease is mediated through the Shh signaling pathway in hepatic stellate cells. Disruption of Shh signaling in the liver injury model led to increased liver T4 compared to liver injury with intact SHH signaling. This could be explained by less T4 to rT3 conversion by Dio3. No changes in liver T3 concentrations were observed. Serum rT3 was also increased after liver injury and returned to baseline after disruption of Shh signaling. To translate these findings to humans, the authors also measured serum thyroid hormone concentrations and liver DIO1 and DIO3 protein expression using immunohistochemistry in patients with mild NAFLD and advanced NAFLD. They found in both a test and validation cohort of human NAFLD, a decreased fT3/rT3 ratio during advanced liver disease accompanied by decreased DIO1 and increased DIO3 protein. It was hypothesized that after liver injury, liver regeneration involving Shh signaling increases *Dio3* expression and decreases *Dio1* expression, leading to less thyroid hormone action. It has not been shown whether inhibition of this pathway decreases regeneration and whether this is a compensatory mechanism or a response to tissue injury. It is, however, known that activation of SHH signaling is involved in several other models of liver disease, including viral hepatitis, cirrhosis, and hepatocellular carcinoma (48). Dio3 is highly expressed at birth and expression decreases with adult age. The upregulation of hepatic Dio3 is observed previously after partial hepatectomy in mouse and rat and might represent a more fetal-like reprogramming to induce regeneration (10). Similar induction of Dio3 expression has been observed after myocardial infarction mediated via hypoxia-inducible factor (HIF)–dependent pathways (49, 50). Although HIF-1 has been shown to be involved in the progression of liver fibrosis, no direct link between HIF-1 and Dio3 in the liver has been investigated (51).

In hepatocellular carcinoma, reduced expression of Dio1 was observed both in the tumors and in the surrounding cirrhotic tissue, although results have been inconsistent (52, 53, 54). No changes in Dio3 expression were observed. Upstream of Dio1, the oncogene astrocyte elevated gene-1 (*Mtda*) and the transcription factor forkhead box A1 (*Foxa1*) play an important role in reducing Dio1 expression in hepatocellular carcinoma (55, 56). Ridruejo and colleagues showed in their HCC model that the decrease in *Dio1* expression was prevented by inhibiting the TGF-β1/SMAD pathway (57). Furthermore, proliferation measured by anti-proliferating cell nuclear antigen was inhibited. Currently, it is unknown whether direct modulation of Dio1 expression alters tumorigenesis in hepatocellular carcinoma. For Dio3, mouse studies have shown changes in microRNAs located in the delta-like 1 homolog (DLK1)-DIO3 genomic imprinted region. Overexpression of this DLK1-DIO3 miRNA cluster positively correlated with HCC stem cell markers. Furthermore, it was associated with a high level of serum α-fetoprotein, a biomarker for liver cancer and for reduced survival rates in HCC patients (58).

Although Dio2 is not measured in adult liver under normal physiology, Kwakkel and colleagues showed that in mouse models of acute and chronic inflammation of the liver increased Dio2 activity can be observed in macrophages (59). Dio2 knockdown in macrophages showed attenuation of an LPS-induced *Il1b* and *Gmcsf* expression, in addition to aberrant phagocytosis. It has been shown that locally produced thyroid hormone is important in macrophage function during inflammation (60). Furthermore, very intriguing results have been obtained for neonatal Dio2 affecting liver disease in adult life. Whereas Dio2 is not measured in adult liver, perinatal hepatic Dio2 expression influences susceptibility to liver steatosis (61, 62, 63). A mouse with hepatocyte-specific Dio2 inactivation (Alb-Dio2KO) exhibits lower liver T3 concentrations at birth. Adult Alb-Dio2KO animals are less susceptible to liver steatosis, hypertriglyceridemia, and obesity when placed on a high-fat diet. Furthermore, Alb-Dio2KO mice did not develop alcohol-induced liver steatosis when given EtOH at an adult age. This is related to low levels of Zfp125, a hepatic transcriptional repressor, in Alb-Dio2KO mice. Zfp125 is increased after EtOH consumption and is known to promote liver steatosis by inhibiting genes involved in the packaging and secretion of very-low-density lipoprotein. Zfp125 is shown to be regulated by an insulin-Foxo1-mediated mechanism and overexpression of Zfp125 leads to hepatic steatosis. These studies show that the fetal programming of the liver by Dio2 influences the susceptibility to liver disease later in life.

## Conclusion and future directions

In this review, we summarized the current studies on hepatic deiodinases and their effects on liver metabolism and disease. It becomes more and more clear that the contributions of liver Dio1 to serum and liver T3 concentrations under euthyroid conditions are found mainly in humans due to higher peripheral conversion and are minor. This could be due to Dio1’s major role in T3 clearance. Therefore, the liver may be important in protecting the body in the hyperthyroid state. Whether the major role of Dio1 on rT3 clearance is liver dependent and clinically significant remains an important question. Stimulation of the thyroid hormone receptor beta (TRβ) using the novel liver-targeted TRβ analog, resmetirom, resulted in significant decreases of serum rT3. This decrease is most likely due to TRβ-mediated stimulation of Dio1 (64). It is clear from all studies that rT3 is a robust marker for Dio1 activity; however, the biological effects of rT3 remain poorly understood.

Regulation of Dio1 and Dio3 has been observed during fasting and a role for Dio1 has been observed during feeding. Although some regulators have been established (leptin, insulin, CAR), the direct regulators of nutrient effects on Dio1 and Dio3 remain largely unknown. Two studies have shown that modulation of Dio1 causes a metabolic phenotype of the liver which did not show a relation to intrahepatic T3 concentrations (31, 33). Whether this is due to the incorrect measurement of T3 or possibly nuclear vs cytoplasmic T3 is unknown. It is intriguing to postulate that Dio1 may have T3-independent effects on metabolism.

It has been shown that Dio2 knockdown in fetal mice protects against liver disease later in life demonstrating that epigenetics can also play a major role in the effects of deiodinases. In liver disease, decreased Dio1 expression and activity has been observed in inflammation, fibrosis, and hepatocellular carcinoma, whereas increased Dio3 has been observed in fibrosis. Whether this is the cause of or compensation for the liver disease remains unknown. For liver steatosis, Dio1 knockdown combined with a WDF leads to increased triglycerides indicating a protective role for Dio1 (31). More studies using Dio modulation are needed to investigate whether the changes are in fact protective or detrimental. This is of interest due to the now-known Dio1 polymorphisms and mutations affecting a significant portion of the population which may alter the course of liver disease. Also, it is of interest whether this may be a target for treating liver disease in the future.

## Declaration of interest

No conflicts of interest for any of the authors.

## Funding

This study did not receive any specific grant from any funding agency in the public, commercial or not-for-profit sector.
